# Modulation of Brain-Derived Neurotrophic Factor (BDNF) Signaling Pathway by Culinary Sage (*Salvia officinalis* L.)

**DOI:** 10.3390/ijms22147382

**Published:** 2021-07-09

**Authors:** Nancy Chiang, Shahla Ray, Jade Lomax, Sydney Goertzen, Slavko Komarnytsky, Chi-Tang Ho, John P. Munafo

**Affiliations:** 1 Department of Food Science, Rutgers University, New Brunswick, NJ 08901, USA; nchiang1208@gmail.com (N.C.); ctho@sebs.rutgers.edu (C.-T.H.); 2 Department of Food Science, University of Tennessee, Knoxville, TN 37996, USA; sray28@utk.edu; 3 Plants for Human Health Institute, North Carolina State University, Kannapolis, NC 28081, USA; jrlomax@ncsu.edu (J.L.); sgoertze16@catawba.edu (S.G.); komarnytsky@ncsu.edu (S.K.); 4 Department of Biology, Catawba College, Salisbury, NC 28144, USA; 5 Department of Food, Bioprocessing & Nutrition Sciences, North Carolina State University, Raleigh, NC 27695, USA

**Keywords:** culinary sage, cognition and memory function, brain-derived neurotrophic factor (BDNF), sage extract, benzyl 6-O-β-D-apiofuranosyl-β-D-glucoside (B6AG)

## Abstract

Culinary sage (*Salvia officinalis* L.) is a common spice plant in the mint family (Lamiaceae) well known for its distinctive culinary and traditional medicinal uses. Sage tea has been used traditionally as a brain-enhancing tonic and extracts from sage have been reported to have both cognitive and memory enhancing effects. Brain-derived neurotrophic factor (BDNF) is an endogenous signaling molecule involved in cognition and memory function. In this study, activity-guided fractionation employing preparative reverse-phase high performance liquid chromatography (RP-HPLC) of culinary sage extracts led to the discovery of benzyl 6-O-β-D-apiofuranosyl-β-D-glucoside (B6AG) as a natural product that upregulates transcription of neurotrophic factors in C6 glioma cells. Purified B6AG showed a moderate dose response, with upregulation of BDNF and with EC_50_ at 6.46 μM. To better understand the natural variation in culinary sage, B6AG was quantitated in the leaves of several commercial varieties by liquid chromatography-mass spectrometry (LC-MS). The level of B6AG in dried culinary sage was found to range from 334 ± 14 to 698 ± 65 μg/g. This study provided a foundation for future investigations, including quantitative inquiries on the distribution of B6AG within the different plant organs, explorations in optimizing post-harvest practices, and aid in the development of sage varieties with elevated levels of B6AG.

## 1. Introduction

Culinary sage (*Salvia officinalis* L.), also referred to by various names including garden sage, kitchen sage, and Dalmatian sage, is a common spice plant in the mint family (Lamiaceae). It is endemic to the coastal areas of Europe, including northern Spain, southern France, and the Balkan Peninsula. Culinary sage has been naturalized in many places and is grown commercially outside of Europe in such countries as India, Japan, South Africa, Brazil, Australia, Canada, and the United States [1].

Sage typically grows well in sunny locations and is commonly propagated from cuttings or seeds [2]. Its cultivars are variable in size, leaf shape, and flower color. While most varieties of sage are primarily used for culinary or medicinal purposes, some varieties have been developed for ornamental purposes and have unique leaf morphology such as large, small, or twisted leaves, growth habits including upright or prostrate habit, as well as different flower or leaf color [3]. The flowers are usually a lavender color, but white, pink, and purple varieties also exist. The two-lipped flowers are formed verticillately on spikes, and bloom in the late spring or summer [1]. The leaf color usually ranges from gray to green, with other colors including purple, yellow, and variegated forms. 

Leaves of culinary sage are consumed either whole or ground, fresh or dried, and added to foods including stuffing, sausage, salads, marinades, and sauces [4]. In addition to its use as a spice, culinary sage has been documented to be used as a general tonic or medicine for its purported healing properties [5]. For example, traditional Iranian herbalists called Attār have been documented using sage to treat or prevent illnesses with knowledge that passes from one generation to the next [6]. The etymology of the word *Salvia* is derived from the Latin word *salvare* meaning ‘to heal’ and *salvere* meaning ‘to be healthy’. The species name, *officinalis*, refers to its traditional medicinal use. Sage tea, which is prepared as an aqueous infusion of dried leaves, has been reported anecdotally to treat various human maladies including gastrointestinal discomfort, mental disorders, menstrual and fertility issues, as well as relieving coughs and sore throats [7]. Although there are documented reports of these uses, a scientific consensus has not been reached on their efficacy [8].

Culinary sage has been used historically for its purported ability to enhance brain function and improve memory. The noun sage is defined as a ‘man of profound wisdom’ while the adjective is defined as ‘wise’. Sage is reported to have antioxidant properties, enhance brain function, improve memory, and delay cognitive decline in adults [9]. Recently, extracts from sage were also reported to have both cognitive and memory enhancing effects in older healthy adults [10]. Although there are reports on the cognitive enhancing effects of sage extracts, the active principles have, thus far, not been determined.

Given the growing interest in the identification of biologically active natural products from plants traditionally used to enhance memory and cognitive function, the objective of the present investigation is to determine if bioactive principles found in culinary sage promote neurotrophic signaling pathways. The specific objectives of the present study are to: (1) employ an activity-guided fractionation approach to identify compounds in dried commercial sage leaves that upregulate neurotrophin expression in a C6 glioma cell line; (2) determine the chemical structure of the active compound(s); (3) perform a dose -response experiment on the purified active compound; and (4) quantitate the active compound(s) in several greenhouse-grown commercial varieties of culinary sage by means of liquid chromatography mass spectrometry (LC-MS). 

## 2. Results

### 2.1. Activity-Guided Fractionation of Sequential Solvent Extracts of Dried Sage Leaves

**Figure 1:**
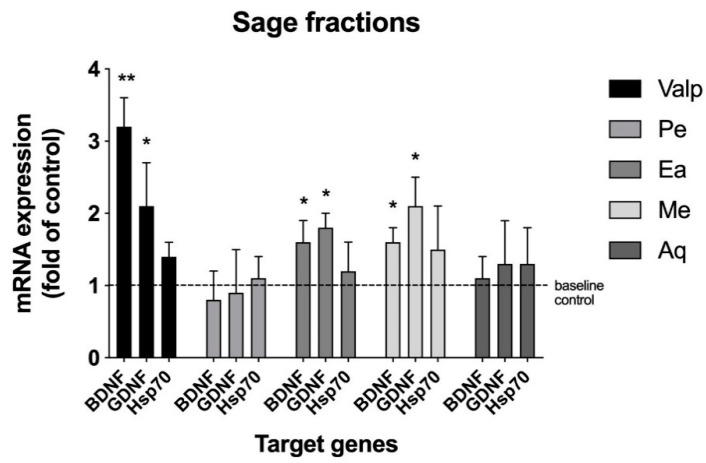
Sage extract modulation of neurotrophin transcription in C6 glioma cells. Sequential solvent fractions Pe (pentane), Ea (ethyl acetate), Me (methanol), and Aq (water) were screened at an initial concentration of 50 μg/mL in triplicate. Results were expressed as a fold change of the control. Valproic acid (Valp) was used as the positive control. * *p* < 0.05, ** *p* < 0.01 versus vehicle control by Tukey’s multiple comparison test.

Dried ground sage leaves were subjected to sequential solvent extraction which resulted in four fractions with increasing polarity, namely, pentane (Pe), ethyl acetate (Ea), methanol (Me), and aqueous (Aq) fractions. The fractions were lyophilized and then screened for modulation of neurotrophic (BDNF: brain-derived neurotrophic factor; and GDNF: glial cell-derived neurotrophic factor) and neuroprotective (HSP70: heat shock protein 70) signaling in C6 glioma cells at an initial concentration of 50 μg/mL. Both the ethyl acetate and methanol fractions showed significant upregulation of BDNF and GDNF mRNAs (approximately 1.5–2.2-fold) (Figure 1). The pentane fraction and aqueous fraction showed no activity in the assay. Since both the ethyl acetate and methanol fractions showed marked activity, the samples were subsequently profiled by LC-MS. Both fractions had similar profiles, albeit different proportions of compounds. The ethyl acetate fraction provided for better chromatographic separation, and therefore was chosen for subsequent preparative fractionation.

**Figure 2:**
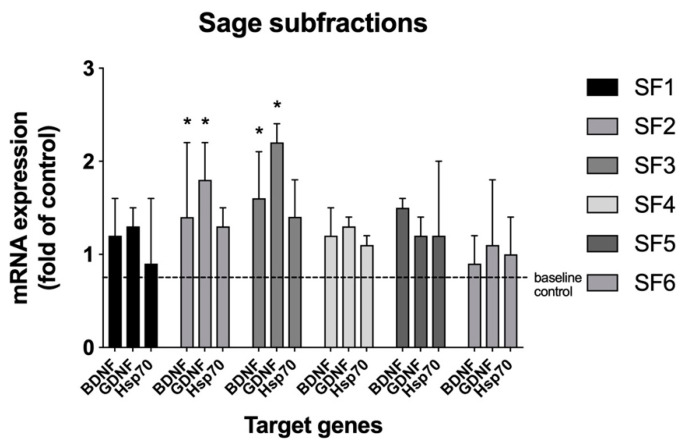
Sage RP-HPLC subfractions SF1 through SF6 modulation of neurotrophic factor mRNA levels in C6 glioma cells. Samples were screened at an initial concentration of 50 μg/mL in triplicate. Results were expressed as a fold change of the control. * *p* < 0.05 versus vehicle control by Tukey’s multiple comparison test.

The ethyl acetate fraction was further fractionated into six subfractions (SF1-6) employing preparative reverse-phase high performance liquid chromatography (RP-HPLC). The resulting subfractions were lyophilized and screened for neurotrophic mRNA modulation in C6 glioma cells. Subfraction SF2 and SF3 both showed significant upregulation of BDNF and GDNF mRNA (approximately 1.4–2.3-fold) in the assay (Figure 2). Due to the overlapping target biological activity, subfractions SF2 and SF3 were pooled together in subsequent chromatographic separations and lyophilized prior to the next fraction step.

**Figure 3:**
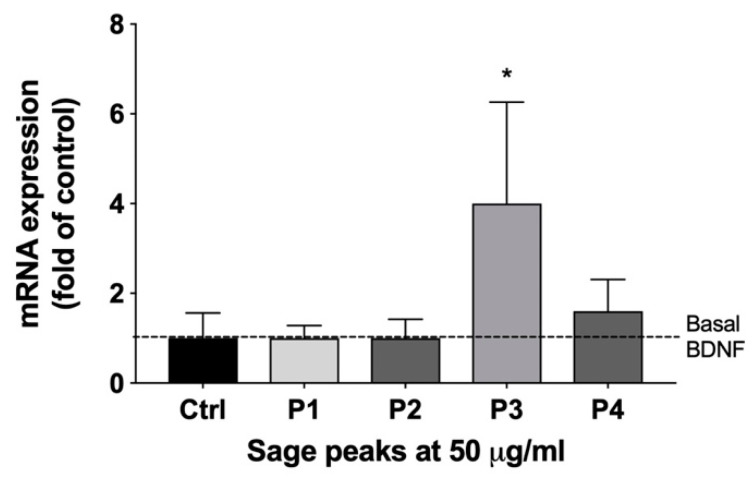
Sage RP-HPLC peak fractions P1 through P4 modulation of BDNF mRNA levels in C6 glioma cells. Samples were screened at an initial concentration of 50 μg/mL in triplicate. Results were expressed as a fold change of the control. * *p* < 0.05 versus vehicle control by Tukey’s multiple comparison test.

Subsequent preparative RP-HPLC was performed on the pooled SF2/3 sample and divided into four different time segments, each corresponding to a predominant peak in the chromatogram (P1–4). Each peak was collected and lyophilized, yielding P1 (5.9 mg), P2 (6.0 mg), P3 (16.9 mg), and P4 (16.6 mg). Since bioactive principles are present in SF2/3 fraction upregulated transcription of both BDNF and GDNF neurotrophic factors but not HSP70 neuroprotective factor, this study was further focused to determine the phytochemical component responsible for upregulating BDNF. Therefore, each P1–4 sample was screened for changes in BDNF mRNA expression in C6 glioma cells. Only sample P3 showed significant upregulation of BDNF mRNA (approximately 4-fold) in the assay. (Figure 3) 

### 2.2. Isolation and Identification of B6AG from SF2/3

**Figure 4:**
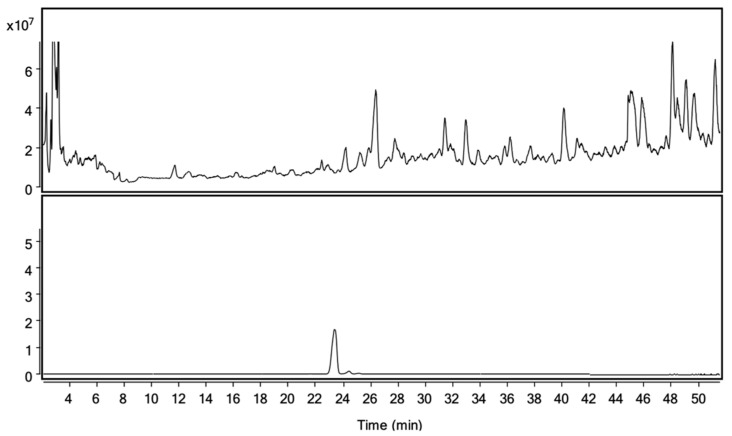
LC-MS total ion chromatogram (TIC) of commercial sage extract (7:3, *v/v*; methanol:water) (top). TIC of purified benzyl 6-O-β-D-apiofuranosyl-β-D-glucoside (B6AG, >90% purity) (bottom).

**Figure 5:**
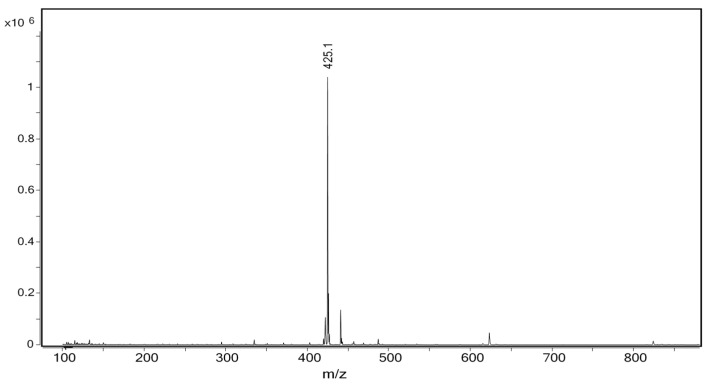
ESI-MS^+^ spectrum of B6AG.

**Figure 6:**
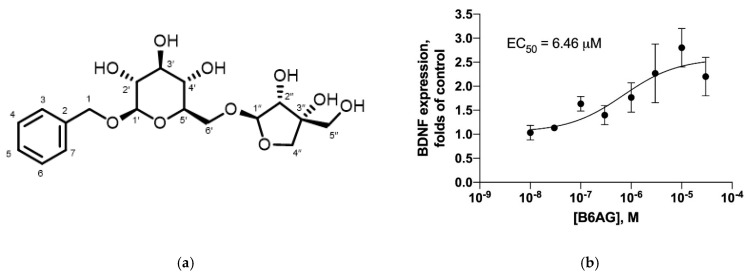
Benzyl 6-O-β-D-apiofuranosyl-β-D-glucoside (B6AG) isolated from sage leaves with BDNF stimulatory activity. (**a**) Chemical structure of B6AG. (**b**) Dose response curve for B6AG upregulation of BDNF mRNA expression.

After successive chromatographic separations, LC-MS (Figure 4), NMR, and a comparison to an authentic standard (Figure 5), we confirmed the identity of the compound in P3 as benzyl 6-O-β-D-apiofuranosyl-β-D-glucoside (B6AG), previously identified in sage leaves (Figure 6a).

### 2.3. Dose Response Curve for B6AG

To gain more insight into the BDNF modulatory activity of B6AG, a dose response experiment was conducted in C6 glial cells at concentrations ranging from 10 µM to 30 mM. The pharmacological activity of B6AG was observed to follow a standard S-shape response with the EC_50_ of 6.46 μM (Figure 6b). 

### 2.4. Quantitation of B6AG in Six Commercial Sage Varieties

**Figure 7:**
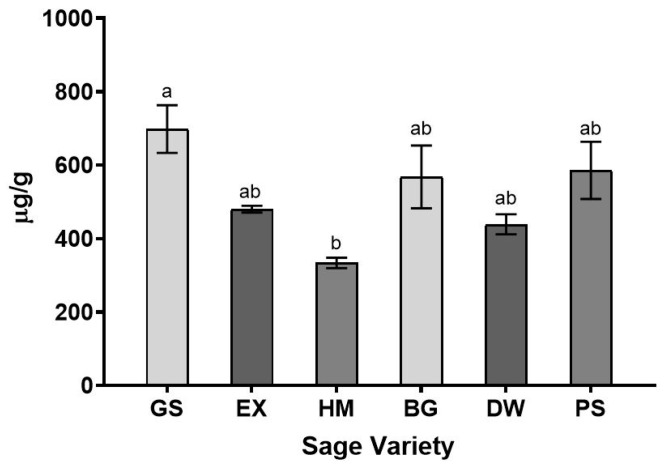
Concentration of B6AG in dried leaves of six culinary sage varieties. Sage varieties are denoted as Garden (GS), Extrakta (EX), Holt’s Mammoth (HM), Berggarten (BG), Dwarf (DW), and Purple (PS). Data are reported as means ± SEM. Samples denoted with the same letter are not statistically distinct.

To gain insight into the natural variation of B6AG in culinary sage, B6AG was quantitated in air-dried leaves of several different greenhouse-grown commercial varieties employing LC-MS. The varieties included Garden, Extrakta, Holt’s Mammoth, Berggarten, Dwarf, and Purple. The level of B6AG in dried culinary sage leaves differed in concentration between the varieties, significantly for Garden and Holt’s Mammoth, at 698 ± 65 μg/g and 334 ± 14 μg/g, respectively (Figure 7).

## 3. Discussion

Neurodegenerative and behavior disorders, especially those related to aging and development of chronic diseases, are closely associated with neurobiological deficits and synaptic pathologies [11,12]. Diverse natural scaffolds that carry multiple chiral centers and functional groups have been evaluated as a potential source of novel pharmacological leads for neurodegenerative disorders, including the modulation of neurotrophic BDNF factor by apigenin, neurotrophic GDNF factor by honokiol, and neuroprotective HSP70 factor by triptolide [13,14,15,16]. 

Neurotrophins are signaling molecules that modulate various biological processes in humans, including neuron development, function, maintenance, and plasticity [17]. Brain-derived neurotrophic factor (BDNF) is a major neurotrophin that regulates neurogenesis, neuronal maturation and survival, and synaptic plasticity, and is hypothesized to be a relevant target to aid in the enhancement of cognition, learning, and memory [18]. It is synthesized as a precursor (proBDNF, 32 kDa) that can be divided into the mature neurotrophin (mBDNF, 14 kDa) or remain unmodified, and then transported to the plasma membrane and released. Regardless of the form, both attach to different receptors with various biological functions. proBDNF binds with high-affinity p75NTR, leading to apoptosis, neurite retraction, and synaptic weakening, and facilitating long-term depression, whereas, mBDNF binds with TrkB receptors promoting cell survival, neurite extension, synaptic strengthening, and long-term potentiation [19]. Given the growing interest in the identification of natural products to enhance cognitive function, the objective of the present investigation was to employ activity-guided fraction of sage extracts to identify compounds in sage that enhance the mRNA expression of neurotrophic and neuroprotective factors, with a primary focus on BDNF, using a C6 glioma cell-based assay.

First, sage leaves were subjected to sequential solvent extraction, resulting in four fractions (pentane, ethyl acetate, methanol, and aqueous) that were screened for BNDF mRNA expression modulation. The pentane and aqueous fractions showed no activity, whereas the ethyl acetate and methanol fractions upregulated BDNF mRNA expression (Figure 1). Since activity was similar in the two fractions, the ethyl acetate fraction was pursued for further fractionation due to its simpler chromatographic profile, as compared with the methanol fraction. The methanol fraction was not further pursued in the current study. Next, larger quantities of the ethyl acetate fraction were generated and subjected to preparative RP-HPLC, affording six subfractions, SF1 though SF6. The subfractions were tested and both the SF2 and SF3 showed activity in the assay (Figure 2). Increased amounts of the two subfractions were pooled together and separated chromatographically by RP-HPLC and were collected using modified time segments corresponding to four predominant peaks in the chromatogram. Four fractions were collected and lyophilized, each containing one of the peaks, denoted as P1 though P4, and subjected to the cell-based assy. When assayed, P3 upregulated BDNF mRNA (4-fold) (Figure 3). The other fractions showed no activity.

Additional quantities of P3 were then purified from larger quantities of sage leaves for characterization (Figure 4). Based on LC-MS, NMR, and chromatographic comparison to a commercially available reference standard, P3 was identified as benzyl 6-O-β-D-apiofuranosyl-b-D-glucoside (B6AG) (>90% purity, as determined by HPLC-DAD-MS) (Figure 5 and Figure 6). B6AG is a known natural product that was originally identified in an Asian plant called large flowered barrenwort, *Epimedium grandiflorum,* and was trivially named icariside F2 [20]. B6AG was also previously identified in a study exploring antioxidant compounds in *S. officinalis;* however, it showed no antioxidant activity in the study [21]. Recently, B6AG was identified in Balinese long peppers, *Piper retrofractum*, and showed moderate α-glucosidase inhibitory activity [22]. In 2015, B6AG was also identified in the leaves of *Eucommia ulmoides.* In that study, it showed anti-inflammatory activity by inhibiting NF-κB at 16.25 ± 2.19 μM [23]. To the best of our knowledge, this present study is the first report of B6AG presented as an active compound in sage leaves that upregulates BDNF mRNA expression. The BDNF upregulating activity of B6AG was observed to follow a standard S-shape response with the EC_50_ of 6.46 mM, which is of a lesser magnitude than the pharmaceutical reference valproic acid (0.5 mM) [24]. This may be related to the diglycoside nature of B6AG and the fact that its neurotrophic activity can be mediated in part by its aglycone, benzyl alcohol. Benzoic acid, a metabolite of benzyl alcohol, was previously described to promote BDNF expression in the effective dose range of 0.25–1 mM [25].

To gain insight into the variation of B6AG levels in different sage varieties, six greenhouse-grown commercial varieties were subjected to quantitative analysis. Significant differences in B6AG levels were only observed between the Garden and Holt’s Mammoth varieties, at 698 ± 65 μg/g and 334 ± 14 μg/g, respectively. No significant differences were observed between the Garden, Extrakta, Berggarten, Dwarf, and Purple varieties and the levels ranged between 439 ± 27 μg/g and 698 ± 65 μg/g. No significant differences were observed between the Extrakta, Holt’s Mammoth, Berggarten, Dwarf, and Purple varieties, and the levels ranged between 334 ± 14 μg/g and 586 ± 78 μg/g. The results of the quantitative experiments suggest that there is some variability in B6AG between sage varieties; however, the overall levels in the varieties tested during this study were low, <0.1% (Figure 7).

In conclusion, the present study employed an activity-guided fractionation methodology which led to the identification of benzyl 6-O-β-D-apiofuranosyl-b-D-glucoside (B6AG) as a natural product from sage leaves that upregulates BDNF mRNA expression in C6 glioma cells. These findings underline the possible application of culinary sage and B6AG as complimentary or adjunct interventions in management of neurodegenerative or depressive disorders. This study also laid the groundwork for future quantitative studies on the distribution of B6AG within the different plant organs (i.e., roots, stems, flowers), studies on optimizing post-harvest practices (i.e., time of harvest, drying), and aided in the development of sage varieties with increased levels of B6AG.

## 4. Methods

### 4.1. Chemicals

The solvents used in this study were obtained commercially and were of HPLC grade. All other chemicals were of analytical grade and purchased from Sigma Aldrich (St. Louis, MO, USA) unless otherwise noted. De-ionized (DI) water (18 MΩ cm) for HPLC separation was prepared inhouse, using a Milli-Q-water purification system (Millipore, Bedford, MA, USA).

### 4.2. Commercial Dried Sage

A commercial sample of USDA organic dried sage leaves (*Salvia officinalis* L.) was obtained from Starwest Botanicals (Sacramento, CA, USA). The indicated origin of the material was Egypt. The dried commercial sample was used for activity-guided fractionation and compound isolation.

### 4.3. Greenhouse Grown Sage Varieties

Six commercial culinary sage cultivars were grown and analyzed during this study. The Extrakta and Garden cultivars were grown from seed; the Berggarten, Dwarf, Holt’s Mammoth, and Purpure were propagated from clones. Both plants and seeds were purchased from Richters Herbs (Goodwood, ON, Canada) and grown at the University of Tennessee (Knoxville, TN, USA). The seeds were planted in professional-grade seed-starting mix in seed trays with clear moisture-retaining covers. After germination, the seedlings were transplanted into 3 in. pots in Pro-Mix (Premier Horticulture Inc., Quakertown, PA, USA) and later transplanted into 1-gallon nursery pots. The cultivars propagated from clones were directly transplanted into 1-gallon pots. The seedlings were maintained in a climate-controlled Venlo-style greenhouse (945 ft^2^) at 25 ± 3 °C with supplemental lighting for 12 h per day. The plants were irrigated daily for 15 min at a rate of 10 mL/min and fertilized biweekly with 20−20−20 water-soluble fertilizer (Southern Ag, Rubonia, FL, USA). The plants were harvested in late July during a period of vegetative growth and air-dried for a minimum of 7 days at 25 °C.

*Garden sage*: This variety grows 16–39” tall and 24–30” wide. It has gray-green leaves that are strongly fragrant. It is commonly used for culinary purposes. 

*Extrakta sage*: This variety grows 24–30” tall and 24–30” wide. It has long gray-green leaves. The plant is used for commercial essential oil production as it contains up to 2.5% essential oil, which is higher than typical concentrations found in other cultivars.

*Holt’s Mammoth sage*: This variety grows 24–30” tall and 30–36” wide. Its leaves are similar to garden sage but are larger in size. This plant grows rapidly, therefore, it is preferred for commercial farmers who must harvest and dry large quantities of leaves.

*Berggarten sage*: This variety is compact and grows 18–24” tall and 30–36” wide. It has gray-green leaves that are wider than most other varieties. This variety has been developed to not produce flowers.

*Dwarf sage*: This variety is compact and grows 15–18” tall and 18–24” wide. It is mostly grown as an ornamental plant.

*Purple sage*: This variety is 18–24” tall and 18–24” wide. It has purple-gray leaves. It is mostly grown as an ornamental plant.

### 4.4. Cell Culture and Gene Expression Analysis

The rat glioma C6 cell line that expresses BDNF was obtained from ATCC (Manassas, VA). Cells were routinely passaged every 3–4 days and maintained in a Dulbecco’s modified Eagle’s medium (DMEM) containing 10% fetal bovine serum (FBS) and 0.1% penicillin–streptomycin at 37 °C and 5% CO_2_. Cells were subcultured into 24-well dishes and, once subconfluent, exposed to a fresh DMEM medium containing a vehicle (0.1% ethanol) or 50 μg/mL of plant extract or bioactive fraction added to a set of three wells per dose. Valproic acid (2 mM) was used as a positive control.

Total RNA was isolated from the scraped cell culture using Trizol reagent (Invitrogen, Carlsbad, CA) and quantified with the SynergyH1/Take 3 spectrophotometer (BioTek, Winooski, VT, USA). The cDNAs were synthesized using 2 µg of RNA for each sample, using a high-capacity cDNA reverse transcription kit on the ABI GeneAMP 9700 (Life Technologies).

Quantitative polymerase chain reaction (qPCR) was performed in duplicate using the following gene-specific primers (IDT, Coralville, IA, USA), GAPDH: forward primer, 5′-CAG TGC CAG CCT CGT CTC AT-3; reverse primer, 5′-AGG GGC CAT CCA CAG TCT TC-3; BDNF: forward primer, 5′-GGC CCA ACG AAG AAA ACC AT-3; reverse primer, 5′-AGG CAC TTG ACT GCT GAG CAT-3; GDNF: forward primer, 5′-GGG ATG TCG TGG CTG TCTT-3; reverse primer, 5′-GTA CAT TGT CTC GGC CGC-3; HSP70: forward primer, 5′-GAG ATC GAC TCT CTG TTC GAG-3; reverse primer, 5′-GCC CGT TGA AGA AGT CCT G-3. Runs were performed on an ABI 7500 Fast (Life Technologies) using 1 cycle at 50 °C for 2 min and 1 cycle at 95 °C for 10 min, followed by 40 cycles at 95 °C for 15 s and at 60 °C for 1 min. The dissociation curve was completed with 1 cycle of 1 min at 95 °C, 30 s at 55 °C, and 30 s at 95 °C. mRNA expression was analyzed using the 2^−ΔΔCT^ method and normalized with respect to the expression of the GAPDH housekeeping genes, using 7500 Fast System SDS Software v1.3.0 (Life Technologies). The amplification of specific transcripts was further confirmed by obtaining melting curve profiles.

### 4.5. Sequential Solvent Extraction of Dried Sage Leaves

Dried commercial *Salvia officinalis* leaves (50 g) were frozen in liquid nitrogen, ground into a fine powder with a coffee grinder, and extracted with pentane (1 × 200 mL followed by 2 × 100 mL). After centrifugation (20,000× *g* for 5 min) (Sorvall RC-3C Plus, Thermo Fisher Scientific Inc., Waltham, MA, USA) and vacuum filtration using a Buchner funnel with a Whatman 114 filter paper (Whatman International Ltd., Maidstone, UK), the supernatant was collected and evaporated under reduced pressure (30 °C; 1.0 × 10^−3^ bar) using a Buchi R-215 rotary evaporator (Buchi, Postfach, Switzerland), yielding the pentane fraction (Pe; 1.06 g). The residue was then extracted with ethyl acetate (3 × 100 mL). After centrifugation (20,000× *g* for 5 min) and vacuum filtration, the supernatant was collected. The supernatant was then evaporated under reduced pressure, yielding the ethyl acetate extract (Ea; 1.49 g). The residue was then extracted with methanol (3 × 100 mL). After centrifugation (20,000× *g* for 5 min) and vacuum filtration, the supernatant was collected. The supernatant was then evaporated under reduced pressure, yielding the methanol fraction (Me; 2.05 g). The residue was then extracted with DI water (3 × 100 mL). After centrifugation (20,000× *g* for 5 min) and vacuum filtration, the supernatant was collected. The supernatant was then evaporated under reduced pressure and lyophilized using a VirTis Advantage XL 70 freeze dryer (SP Scientific, Warminster, PA, USA), yielding the aqueous fraction (Aq; 6.60 g). The samples were then stored at −80 °C until further analysis.

### 4.6. Preparative Reverse-Phase High-Performance Liquid Chromatography RP-HPLC

Fractionation of ethyl acetate extract was achieved by semipreparative RP-HPLC performed on a Gemini C18 column (250 mm × 21.2 mm i.d.; 5 μm particle size) (Phenomenex) to afford subfractions SF1–6. Chromatography was performed on an Agilent 1260 Infinity II high-performance liquid chromatograph (Agilent Technologies, Santa Clara, CA, USA) using a UV-Vis detector and a 2 mL injection loop. A 1260 Infinity II fraction collector (Agilent Technologies, Santa Clara, CA, USA) was used to gather the fractionated material. Mixtures of (A) 0.1% formic acid in deionized water and (B) 0.1% formic acid in methanol were used as the mobile phases. The flow rate was set to 20 mL/min, the column temperature was 23 ± 2 °C, and UV detection was recorded at λ = 280 nm. The ethyl acetate extract was dissolved in a mixture of mobile phase A and mobile phase B (75:25, *v/v*) and filtered through a 0.45 μm PTFE syringe filter prior to injection. Chromatography was performed using a linear gradient of 5–90% B over 65 min, and then held at 90% B for 10 min. The re-equilibration time was 10 min. The target subfractions SF1–6 were collected at the following time segments: SF1, 8–14 min; SF2, 14–20 min; SF3, 20–27 min; SF4, 27–35 min; SF5, 35–44 min; and SF6 44–50 min. The eluent was collected at each time segment and then freed from solvent under reduced pressure (30 °C; 1.0 × 10^−3^ bar), lyophilized, and stored at −80 °C until used for the cell culture experiments or additional fractionation. After the results of the cell-based assay, subfractions SF2 and SF3 were pooled together and further fractionated into fractions P1 through P4, employing the same method as described above, but collected at different time segments. The fractions P1–4 were collected at the following time segments: P1, 14–15.5 min; P2, 15.5–17 min; P3, 17–23 min; and P4, 23–24.5 min. The eluent collected at each time segment was then freed from the solvent under reduced pressure (30 °C; 1.0 × 10^−3^ bar), lyophilized, and stored at −80 °C until further analysis.

### 4.7. Liquid Chromatography-Mass Spectrometry (LC-MS)

LC–MS analyses of *S. officinalis* extracts and pure compounds were performed with an Agilent 1260 series HPLC system interfaced with a 6410 triple-quadrupole LC–MS mass selective detector with an API-ESI ionization source (Agilent Technologies Inc., Santa Clara, CA, USA). The system was equipped with an autosampler, a BIN Pump SL binary pump, a TCC SL thermostatted column compartment, and a DADSL diode array detector. Chromatographic separations for 10 μL injection volumes were performed using a Gemini column (250 × 4.6 mm i.d.; 5.0 μm particle size) (Phenomenex, Torrance, CA, USA). The column temperature was set at 25 °C and operated at a 1.0 mL/min flow rate. DI water with 0.1% formic acid (A) and methanol with 0.1% formic acid (B) were employed in the binary mobile phase with a linear gradient of 5–55% B for 50 min, 55–90% for 5 min, and elution at 90% for 5 min, followed by re-equilibration for 10 min. Data acquisition and analyses were performed using Mass Hunter Workstation Data Acquisition, Qualitative Analysis, and Quantitative Analysis software. LC–MS analysis was performed in both negative and positive ion mode with ionization parameters set at: capillary voltage, 3.5 kV; nebulizer pressure, 35 psi; drying gas flow, 13.0 mL/min; drying gas temperature, 350 °C; and mass scan range, m/z 300–2000.

### 4.8. Confirmation of B6AG

Benzyl 6-O-b-D-apiofuranosyl-b-D-glucoside (B6AG). Amorphous powder; C_18_H_26_O_10;_ ESI^+^-MS, *m/z* 425.1 (100, [M+Na]^+^) (Figure 6); ESI^−^-MS, *m/z* 401.3 (100, [M-H]^−^); ^1^H NMR (400 MHz) δ_H_ 3.26 (1H, dd, J = 7.9, 8.3 Hz, 2′-H); 3.31 (1H, m, 4′-H); 3.34 (1H, m, 3′-H); 3.42 (1H, m, 5′-H); 3.62 (2H, s, 5″-H); 3.65 (1H, dd, J = 6.3, 11.4 Hz, 6′-H); 3.80 (1H, d, J = 10.2 Hz, 4″-H); 3.97 (1H, d, J = 2.0 Hz, 2″-H); 4.02 (1H, d, J = 10.2 Hz, 4″-H); 4.03 (1H, dd, J = 1.5, 11.4 Hz, 6′-H); 4.35 (1H, d, J = 7.9 Hz, 1′-H); 4.68 (1H, d, J = 12.0 Hz, 1-H); 4.91 (1H, d, J = 12.0 Hz, 1-H); 5.07 (1H, d, J = 2.0 Hz, 1″-H); 7.35 (2H, t, J = 7.2 Hz, 4/6-H); 7.45 (2H, d, J = 7.2 Hz, 3/7-H); 7.45 (1H, t, J = 7.2 Hz, 5-H); ^13^C NMR (400 MHz) δ_C_ 64.6, 67.2, 70.6, 70.8, 74.1, 74.2, 76.0, 77.0, 79.5, 102.0, 109.9, 127.9, 128.2, 128.3, 137.9; samples were dissolved in CD_3_OD; d_C_ = 48.0 ppm; d_H_ = 3.33 ppm. The ^1^H and ^13^C NMR data for benzyl 6-O-b-D-apiofuranosyl-b-D-glucoside (icariside F2) was consistent with the literature [20,21].

### 4.9. Preparation of Analytical Standards

B6AG, isolated and purified as described above, was used to prepare analytical standards. The compound was weighed into volumetric flasks (10 mL), dissolved in methanol and DI water (7:3, *v/v*; 1 mL), and sonicated for 5 min, and then taken to full volume. Standard equations were calculated using three data points. To establish calibration equations, mean peak areas (n = 3) from the extracted ion chromatogram (EIC) (m/z 425) generated from each standard solution were plotted versus concentration of each standard. Standard solutions were stored at 4 °C and equilibrated to ambient temperature before use.

#### 4.9.1. Sample Preparation for Quantitative Analysis

To determine the level of B6AG, several different sage varieties were evaluated through quantitative LC–MS analysis. Material (0.2 g) was dissolved in 3 mL solution (7:3, MeOH: DI water *v/v*), vortexed for 1 min, and sonicated for 20 min. The solution was centrifuged, then, after the supernatant was collected, was filtered with a 0.45 μM PTFE filter (Whatman, PA, USA) prior to LC-MS analysis.

#### 4.9.2. Quantitative Analysis

Quantitative analysis was performed by LC-MS in positive ion mode using the extracted ion (m/z 425) with the same ionization parameters as described above. Linear relationships between ion area and standard concentrations showed good linearity over the concentration range of 75−600 μg/mL. The correlation coefficient was R^2^ = 0.9997. Recovery rate experiments (n = 3) were performed and were calculated (94%) for B6AG in the sage powder using the standard addition method [26].

### 4.10. Nuclear Magnetic Resonance Spectroscopy (NMR)

^1^H and ^13^C spectra were acquired with a 400 MHz liquid state NMR spectrometer (Bruker Corporation, Billerica, MA, USA). Data was analyzed using Bruker TOPSPIN 4.0.9 software. Samples were dissolved in CD_3_OD. Chemical shifts were calculated as δ values with reference to TMS, and J values were calculated in Hz.

### 4.11. Statistical Analysis

Data were analyzed by one-way analysis of variance (ANOVA) followed by Tukey multiple-range tests using Prism 8.0 (GraphPad Software, San Diego, CA, USA). All data were presented as means  ±  SEM. Significant differences were accepted when the *p*-value was < 0.05. For the quantitation experiments, all analyses were performed on individual plants in at least triplicate. Tabulated data were analyzed by use of one-way ANOVA (α < 0.05). Means were separated with the Tukey–Kramer HSD test (α < 0.05) using JMP Pro 20.0 software (SAS Institute, Cary, NC, USA).

## Data Availability

Data is available from the corresponding author upon request.
